# Unmet needs and gaps in the identification of secondary progression in multiple sclerosis: a Southern Italy healthcare professionals’ perspective

**DOI:** 10.1007/s10072-022-06402-3

**Published:** 2022-09-17

**Authors:** Giacomo Lus, Marco André Bassano, Vincenzo Brescia Morra, Simona Bonavita, Antonio Gallo, Davide Maimone, Laura Malerba, Giorgia Teresa Maniscalco, Francesco Saccà, Giuseppe Salemi, Renato Turrini, Salvatore Cottone, Edoardo Sessa, Maria Buccafusca, Luigi Maria Edoardo Grimaldi

**Affiliations:** 1 Department of Advanced Medical and Surgical Sciences, II Division of Neurology, Multiple Sclerosis Center, University of Campania “L. Vanvitelli”, Naples, Italy; 2 Novartis Farma S.P.A, Origgio, VA Italy; 3 Department of Neurosciences Reproductive Sciences and Odontostomatology, Multiple Sclerosis Center, Federico II University, Naples, Italy; 4 Department of Advanced Medical and Surgical Sciences, Università Della Campania Luigi Vanvitelli, Naples, Italy; 5 Unità Operativa Complessa Neurology, Multiple Sclerosis Center, ARNAS Garibaldi, Catania, Italy; 6 Multiple Sclerosis Center, “A. Cardarelli” Hospital, Naples, Italy; 7 UOC of Neurology and Multiple Sclerosis Center, DAI of Diagnostic and Interventistic Radiology and Stroke, AOIP “P. Giaccone”, Palermo, Italy; 8 Neurology and Stroke Unit, Multiple Sclerosis Center, ARNAS CIVICO, Palermo, Italy; 9 IRCCS Centro Neurolesi “Bonino-Pulejo”, Messina, Italy; 10 Neurology and Neuromuscular Unit, Multiple Sclerosis Centre, “G. Martino” University Hospital, Messina, Italy; 11 Neurology and Multiple Sclerosis Center, Unità Operativa Complessa (UOC), Foundation Institute “G. Giglio”, Cefalù, PA Italy

**Keywords:** Multiple sclerosis, Secondary progressive multiple sclerosis (SPMS), Biomarkers, Diagnosis, Italy, Expert opinion

## Abstract

**Objective:**

Multiple sclerosis (MS) is a chronic disease with different clinical courses and a tendency to worsening. The relapsing–remitting MS presents acute onset and relapses of neurological symptoms, followed by their remission. This form can convert to secondary progressive MS (SPMS) with irreversible neurological worsening and disability. The identification of signs, symptoms, markers of progression, and strategies to manage MS patients is mandatory to allow early identification of those at higher risk of conversion to SPMS, for prompt intervention to cope with the progression of the disease.

**Methods:**

A panel of Italian experts from Southern Italy have reviewed the current knowledge on MS and its management and identified the crucial tools for SPMS recognition.

**Results:**

More effective communication between patients and clinicians should be established, with the support of digital tools. Moreover, the improvement in the clinical use of biomarkers for progression (cellular structures and tissue organization, such as neurofilaments and chitinase 3-like 1, axonal and neurons density) and of instrumental analyses for recognition of whole-brain atrophy, chronic active lesions, spinal cord lesions and atrophy, and the improvement the combination of the Expanded Disability Status Scale and the evaluation of cognitive dysfunction are discussed.

**Conclusion:**

Given the availability of a pharmacological option, adequate education both for patients, regarding the evolution of the disease and the specific treatment, and for professionals, to allow more effective and sensitive communication and the best use of diagnostic and management tools, could represent a strategy to improve patient management and their quality of life.

## Introduction

Multiple sclerosis (MS) is a highly heterogeneous chronic disease characterized by a continuum of disease phenotypes, with a tendency to worsening. It is estimated that around 2.3 million people live with MS worldwide, with a higher prevalence in women [1]. The onset of MS is common in young adults, although pediatric and late-onset disease also occurs [1].

The different clinical courses have been classified according to disease activity and disability progression [1, 2], which are evaluated with an objective scale, the Expanded Disability Status Scale (EDSS) [3].

In 85–90% of MS patients, the disease is characterized by acute onset and relapses of neurological symptoms, followed by their remission, the so-called relapsing–remitting MS (RRMS) [4]. About 50–80% of patients experience conversion from RRMS to the secondary progressive multiple sclerosis form (SPMS) within 15–20 years from diagnosis [4]. SPMS is characterized by irreversible worsening of neurological symptoms and disability, irrespective of relapses [5]. Despite the numerous disease-modifying treatments (DMT) available for RRMS, the conversion to SPMS continues to be a frequently observed phenomenon. Early identification of RRMS patients at higher risk of conversion could allow the clinicians a more effective use of the available DMT. However, there is a lack of simple and reliable tools in clinical practice to allow the rapid identification of subjects at high risk of transition to SPMS [6]. Early and effective treatment of RRMS may help to tackle the progression of MS, as demonstrated by the study of Brown et al. on the effectiveness of early (i.e., within 5 years of disease onset) treatment of RRMS on conversion to SPMS [7].

Identifying the right timing of the transition from RRSM to SPMS is of pivotal importance for adequate and timely treatment, given the availability of an effective and safe drug for its active form, siponimod, which was approved by the US Food and Drug Administration (FDA) in March 2019 [8] and by the European Medicines Agency (EMA) in January 2020 [9]. The accurate identification of disease status is critical because patients with SPMS are also likely to require additional physical and cognitive rehabilitation and psychological treatment to cope with the long-feared progression of the disease [10, 11].

Despite the well-characterized neuropathological features of SPMS (extensive and confluent demyelinating lesions together with chronically active-expanding lesions throughout the brain and spinal cord [12, 13] and meningeal inflammation and white and gray matter atrophy [14]), the early recognition of SPMS patients is a paramount challenge for healthcare professionals (HCPs). This is due to the following: (i) the lack of diagnostic tests, standardized protocols, and criteria to identify the turning point leading from RRMS to SPMS [15, 16], (ii) the absence of specific symptoms indicating the progression [17], and (iii) the difficulties in reaching an effective interaction between the MS patient and the physician, often delaying the recognition of the transition phase. Indeed, due to the slow and insidious progression of the transition phase to SPMS, the worsening is not always evident in terms of EDSS scores and can go unnoticed both by patients and HCPs. Progression from RRMS towards SPMS can be masked by inflammatory processes, relapses, treatments, and compensatory mechanisms deserving the definition of “silent progression” [18]. Aging is another important risk factor for progression that should be considered, especially in elderly patients and in those with a late onset of the disease.

All of the abovementioned factors and difficulties in identifying early transition to SPMS contribute to the underestimation and delay of the diagnosis. Indeed, identification of SPMS patients is mostly retrospective, thus leaving a long period of uncertainty [17, 19].

In the present work, we review the current needs, gaps, and criticisms in SPMS and discuss the possible approaches suggested by a panel of experts in Italy, to implement the effective identification and management of SPMS.

## SPMS therapies

The lack of a specific treatment for SPMS has been the main critical point in disease management. The available RRMS therapies may delay progression/conversion to SPMS by reducing brain damage, although evidence is conflicting [20]. The first DMT approved for the treatment of SPMS was mitoxantrone [21]. The efficacy of mitoxantrone is due to a broad mechanism of action, from decreasing the secretion of proinflammatory cytokines to inhibition of macrophage-mediated myelin degradation [22]. This type 2 topoisomerase inhibitor acts on neuroinflammation and has been shown to reduce the number of relapses and time to first relapse and improve EDSS and the ambulation index [21]. Its use is limited by unfavorable adverse effects, i.e., cardiotoxicity [23], hepatotoxicity, and hematologic malignancy [24]. Mitoxantrone remained the only FDA-approved drug for the treatment of SPMS until 2019.

Interferon-beta-1b is also approved for the treatment of SPMS, as a result of its antiviral, anti-inflammatory, antiproliferative, and immunomodulatory activities, although a 10-year follow-up of the European trial testing and its efficacy in SPMS patients did not convincingly support its long-term use [25].

Different clinical trials have investigated the efficacy and safety of other approved MS treatments on progressive MS. Clinical studies focusing on the efficacy of anti-CD20 antibodies (rituximab and ocrelizumab) and S1P receptor modulators (fingolimod and siponimod) enrolled primary progressive MS (PPMS) and SPMS patients. B-cell-depleting therapy with rituximab or ocrelizumab showed some benefits for the treatment of young PPMS patients with inflammatory lesions, slowing disease progression up to 120 weeks [26, 27]. Despite PPMS and SPMS sharing some pathophysiological features, benefits from the treatment with rituximab and ocrelizumab have not yet been demonstrated in a representative SPMS population. As regards other RRMS-approved DMTs, fingolimod did not reduce disease progression in PPMS in the INFORMS phase 3 clinical trial [28], while natalizumab failed to show an improvement based on the EDSS scale in SPMS patients in the ASCEND phase 3 trial [29].

The most encouraging results for the treatment of SPMS came from the EXPAND study, a phase 3 RCT investigating the role of siponimod in delaying disability progression. In this study, siponimod, a more selective modulator of the S1P receptor than fingolimod, showed that intervention reduced the relative risk of 3-month and 6-month confirmed disability progression (CPD) by 21% (*p* = 0.01321) and 26% (*p* = 0.0058), respectively [30]. Moreover, pre-defined patient’s subgroup analysis of the primary endpoint (3-month CDP) indicated greater efficacy of siponimod in younger patients, with lower EDSS and shorter disease duration (i.e., baseline age of 20–30 years, EDSS score at baseline ≤ 5, and duration of MS since first symptoms of 10 years — early phase SPMS) [30]. The analysis of the secondary endpoint (6-month CDP) in the overall population and in specific subgroups defined by relapse activity and disease progression supported this finding [30]. Siponimod is now approved and marketed for the therapy of SPMS with active disease as evidenced by relapses or imaging features of inflammatory activity. No DMTs have so far been approved for the treatment of the inactive form of SPMS, even if the recent 2021 update of the European Academy of Neurology (EAN)/European Committee for Treatment and Research in Multiple Sclerosis (ECTRIMS) guidelines suggests the use of siponimod both in patients with active and non-active SPMS [31].

Research and clinical studies have been performed in the past, and are still ongoing, to target, among others, molecular mechanisms involved in MS progression, such as oxidative stress [32], brain atrophy [33], demyelination [34], and lack of remyelination [35].

## Physician–patient interaction

The unavailability of safe and effective therapies for SPMS often causes the discontinuation of DMTs in patients with RRMS experiencing disability progression, as patients and caregivers deal with the difficulties associated with the transition awareness process and communication with physicians [36].

The reactions to the communication of progression vary greatly across patients. In some cases, the patient realizes that the disease is slowly progressing even before the physician does. Indeed, a recent study demonstrated that patients realize SPMS conversion on average 2.7 years before the confirmation from the neurologist [37]. Some patients accept the progression, while others experience a denial phase towards identifying SPMS [36], ignoring the availability of a specific treatment and fearing potential loss of motor function. Some patients may be reluctant to share information related to worsening of symptoms because they fear there will be no chance of further treatment [36].

To avoid anxiety, fear, and delay in the intervention, timely and sensitive communication between physicians and patients is paramount [36]. Technical terms related to the pathology may generate confusion among patients, who should be able to express their concerns about their everyday life without hesitancy in discussing progression issues [36]. The timing of information delivery should be carefully considered by the physician; communication soon after the diagnosis may imply a negative reaction by the patient, but postponing the topic would reduce the acceptance of the situation and might fail to engage the patient in the treatment positively.

Communication of shifting into the progressive phase should always be accompanied by the perspective of the newly available treatments [36]. Defining the disease as a continuum, more than as a sequence of phases, using patient-friendly language, might help to increase patient-physician trust, creating awareness towards the pathology and encouraging engagement in the treatment.

The ManTra (Managing the Transition to SPMS) study was a mixed-method research project conducted in Italy and Germany to gather insights into the patient perspective concerning the transition period. The project used an online survey to assess the experience of patients newly identified with SPMS, verifying their needs and exploring patients’ perspectives about their transition phase to SPMS. The results of the project showed that around 40% of patients were not aware of their progressive condition [37]. This situation is particularly common in the southern regions of Italy, thus indicating that sociocultural biases influence the phenomenon, and that the necessary improvement of the physician-patients communication must be tailored to different geographic areas.

## Biomarkers for progression identification

Some prognostic factors predicting transition to SPMS have been identified. Lesions in the gray matter, spinal cord, and infratentorial regions and high inflammatory activity at the disease onset and in the following 5 years are well-known risk factors for future progression [38]. Thus far, biological markers with a reliable prognostic value are missing and are definitively needed. Here, we report the most promising biomarkers deserving attention in the research settings, which still need to be validated through standard procedures before implementation into routine clinical practice.

### Neurofilaments and chitinase 3-like 1

Neurofilament light chain (Nfl), a cytoskeletal polypeptide of the neuronal axon [39] from cerebrospinal fluid and serum, can be accurately measured by single-molecule array (SIMOA) technology. The procedure is sensitive and reproducible, allowing the definition of a value that correlates well with EDSS score and disease activity and progression. Some limitations hamper 45 Nfl use as a biomarker, including that its increase is not specific for MS, as it indicates axonal destruction common to other neurodegenerative pathologies. Moreover, blood levels of Nfl rise with aging and are affected by disease activity, treatments, and comorbidities [40].

Chitinase 3-like 1 (CHI3L1) is a glycoprotein secreted by activated glia, whose increment has been related to disease progression, cognitive impairment, and disability [41]. However, serum CHI3L1 levels are not specific for MS, and their evaluation should be done by the analysis of the cerebrospinal fluid, which is difficult in the clinical setting. Measuring Nfl and CHI3L1 may help in identifying the subset of RRMS patients that will experience progression, but the invasiveness of evaluating CHI3L1 strongly limits its determination [41].

### Retinal nerve fiber layer and ganglion cell/inner plexiform layer thickness

MS often affects the anterior visual pathway; thus, the retina represents a unique anatomical window to directly study neuronal damage. Retinal nerve fiber layer (RNFL) [42], reflecting the axonal density, and ganglion cell/inner plexiform layer thickness (GCIPL) [43], reflecting the density of neurons, can be measured by optical coherence tomography (OCT). RNFL and GCIPL have been shown to correlate well with increased risk of disability progression, although they show individual variability [44].

### PET radiotracers uptake

The uptake of PET radiotracers that bind translocator protein (TSPO)-like ^11^C-PK11195 [45], ^11^C-PBR28 [46], and ^18^F-PBR06 [47], expected to mirror microglia activation, is higher in SPMS patients. The use of PET, mostly limited to research settings and still to be implemented in clinical practice, shows high potential in MS, as it would allow the targeting of many relevant mechanisms involved in demyelination, neuroinflammation, and neurodegeneration [48]. The use of PET in clinical practice is limited by the fact that TSPO ligands are not specific for MS, by the lower spatial resolution of the technique compared to MRI, and by safety concerns regarding ionizing radiation [49].

### MRI biomarkers

Radiological biomarkers of progression are also employed more widely in research than in clinical practice, and a standardized method for the application of MRI is lacking. MRI detection of whole-brain atrophy (WBA) is a strong predictor of disability progression that could also help to discriminate stable patients from those in silent progression, as the latter tend to have a higher rate of brain atrophy [50]. However, WBA measure can be affected by fluctuations in brain volume due to internal (e.g., age, metabolism) and external (e.g., fluid intake, drugs, alcohol consumption) factors [51]. WBA has been incorporated in the “no evidence of disease activity-4” (NEDA-4) criteria already including the more diffuse NEDA-3 criteria, clinical markers such as acute relapses and sustained disability progression, and one conventional MRI marker (i.e., gadolinium-enhancing lesions or new/enlarging T2 lesions) [52].

Selected/specific MRI markers known to be involved in MS progression have gained considerable attention over the past years. However, advanced MRI techniques are necessary to detect some relevant biomarkers, such as cortical and deep gray matter damage, which are significantly associated with disease progression [53]. Contrariwise, ventricular enlargement, an indirect marker of WBA, can be more easily measured and has been shown to correlate with disease progression [54].

More recently, great interest has been solicited by the identification of chronic active lesions (CAL, also called “slowly expanding” or “smoldering” lesions). These are characterized by accumulation of microglia and macrophages at the edge of the lesion, dysfunction of the blood–brain barrier, and more severe demyelination and axonal injury [55]. CALs expand over time and have a detrimental effect on the clinical course of the disease, such that their number has been shown to correlate well with disease and disability progression [55]. Unfortunately, CALs can be visualized only using high-field MRI scanners (operating at 3 T [56] and 7 T-MRI [57]), appearing as lesions with paramagnetic hypointense rim, due to the presence of iron-laden-activated microglia. The presence of activated microglia at the edge of CALs also allows their identification with PET tracers (i.e., C^11^-PK11195) [58]. CALs represent a new potential/valuable biomarker to predict the transition to SPMS and to evaluate the effect of DMTs on the development and evolution of these detrimental lesions.

Other recently proposed brain MRI markers of disease progression are represented by (i) the *atrophied lesion volume*, which is the volume left by atrophied T2 lesions replaced by cerebrospinal fluid and which might be an earlier predictor of progression compared to WBA [59], and (ii) *leptomeningeal inflammatory infiltrates* [60], which need delayed high-resolution postcontrast T2-FLAIR MRI in order to be identified.

Spinal cord (SC) lesions as well as measures of SC atrophy have also been found to be highly predictive of MS progression, but SC images, particularly those suitable for atrophy measurements, are not yet routinely acquired. This limit could be overcome by including the cervical portion of the spinal cord into the standard acquisition MRI protocol of MS patients [50].

## Identifying patients transitioning towards SPMS: models and tools

Developing models to identify MS patients at risk of progression has been the focus of many studies. Manouchehrinia and colleagues generated a nomogram showing high predictive value for the risk of progression to SPMS over 10–20 years, based on year of birth, sex, age at onset, EDSS score, and age at the first EDSS score [61]. Another study employed both quantitative and qualitative approaches through a multivariate analysis of observational study data that identified discriminating factors between RRMS and SPMS arising from interviews with patients and HCPs that explored impacts associated with transition [17]. These tools would provide support to clinicians in everyday practice, without substituting the clinician experience and the need for instrumental examinations.

The MS Progression Discussion Tool (MSProDiscuss) is another model created by clinicians to facilitate interactions between HCPs and patients and to get insights into the patient’s perspective of MS. It includes quantitative and qualitative assessments and may help close monitoring of patients’ symptoms and impacts during transition [62]. However, it still needs to be validated in routine clinical practice.

### The EDSS scale

The scales and scores used thus far to measure MS-associated disability need implementation to increase their accuracy. The EDSS and the pyramidal functional system score are the most widely standardized tools in both research and clinical practice. However, the EDSS has shown limited accuracy, as it has proven to be relatively insensitive in detecting upper extremity and cognitive dysfunction. Moreover, it overestimates long-distance ambulation and does not allow proper measurement of short-distance ambulation. It should also be considered that ambulation abilities are often self-reported by patients, thus decreasing the accuracy of the final EDSS score.

A newer endpoint, EDSS plus, defined as 24 weeks confirmed worsening of any of these three components (EDSS, lower-extremity function measured by timed 25-foot walk and upper-extremity function, measured by 9-Hole Peg Test) is likely to be more sensitive than EDSS alone in measuring disability progression in transition to SPMS [63]. Also, the integration of the EDSS with cognitive measures, such as the Brief International Cognitive Assessment for MS (BICAMS), could increase the accuracy of the measure [64].

### Cognitive dysfunction

Cognitive dysfunction is a common accompaniment of SPMS, with a very high prevalence in this clinical phenotype [65]. Usually, language abilities are relatively spared, while information processing speed (IPS) and episodic memory are domains more heavily affected [66]. There are no formal guidelines regarding the assessment of cognitive functions in MS. However, an Italian expert panel meeting provided recommendations such as the regular assessment of the cognitive functions every 6 months, which, in addition to the EDSS score, would be of value for the early identification of secondary progression and the monitoring of SPMS patients [67].

Some studies have focused on developing digital tools for evaluating cognitive functions in MS. A computerized version of the Symbol Digit Modalities Test (cSDMT) was developed to evaluate information processing speed, a frequently observed cognitive deficit in MS patients, in an easier and more reproducible way [68]. cSDMT can be used as an app on smartphones allowing a closer follow-up of symptoms. A key of 9 symbol-digit pairs is provided during the test to the patient, who then has to associate the correct numbers to the symbols within 90 s [69]. The advantage of the digital version compared to the classical test is the random changes of the key supported by the app, thus avoiding the outcome being affected by code memorization (training effect) after repeated testing [69].

SDMT can be combined with both MRI and the EDSS score to evaluate the effect of treatment. An oral version of the SDMT, which does not require the need to hold a pen or to touch the screen, has also been developed for patients with severe upper arm impairment [69].

The BICAMS adds to the SMDT a visuospatial test (Brief Visuospatial Memory Test – Revised [BVMT-R]) and a verbal memory assessment (California Verbal Learning Test). International validation exists for numerous countries and takes approximately 15 min. It gives a more accurate neuropsychological assessment than the SDMT and can be easily integrated with the EDSS. This results in a more accurate final score calculation in 25% of patients [64].

### E-health: telemedicine and digital diagnostic tools

Telemedicine is currently seen as one of the best tools that will help the prompt identification of changes in the disease course by allowing frequent physician–patient interaction. A comprehensive neurological examination requires 40–60 min on average for each patient, and reducing the time for patient-neurologist encounters could lead to inaccurate diagnosis and poor patient outcomes. A Virtual Visit Assessment (ViVA) following a structured protocol has been suggested for virtual patient management [70]. This protocol, successfully tested during the COVID-19 health emergency, is composed of five steps (including pre-visit with a sharing documents phase, a virtual visit, and a post-visit phase), takes around 54 min to complete and actively involves the caregiver. Besides the potential benefits in terms of reduced contagion risk, the ViVA model encountered the satisfaction of the patients, allowing the simplification of visit logistics, the re-enforcement of the link between patient and physician while resulting in a limitation of overall costs [70], thus being a valid alternative to the established standard visit.

Moreover, the aforementioned MSProDiscuss tool is reported to be reliable, simple, sensitive, and with good specificity; the neurologists confirmed its usability in clinical practice, and it can therefore represent another valid digital tool for patient evaluation in the absence of the in-person visit [62].

One possible drawback of the use of telemedicine and digital tools is that they can be limited in older patients, who could lack Internet access or may not be supported by the appropriate device. Besides, limited Internet access and a lack of Wi-Fi connection may be shared issues for several patients. HCPs should carefully consider these factors before suggesting the routine use of a digital app.

## Expert opinion

Given the delayed detection and the lack of standardized tools to identify patients, the SPMS population in Italy is likely to be underestimated. A panel of 12 Italian MS experts, six from the Campania region and six from Sicily, took part in two virtual round tables (due to COVID-19 policy restrictions), to discuss the needs and the gaps in SPMS identification and management in this area of Italy and the possible ways to anticipate diagnosis and start the appropriate treatment promptly. Such issues, although with slight differences among the different regions, are common to the whole country. Board members were selected within these regions giving the proximity of the areas and the similarity in clinical and patient care organization. Inclusion criteria for the healthcare professionals were the 10-year experience in clinical neurology, their involvement in MS clinical research and patient treatment, and their publishing record related to MS.

Before the first meeting, the participants reviewed the literature with a particular focus on the most recent publications (from 2019 to 2021) searching PubMed with specific keywords (Multiple Sclerosis progression AND patient-reported outcome OR patient communication; Multiple Sclerosis progression — all fields AND biomarkers — Title and Abstract; Multiple Sclerosis progression AND diagnostic tools), concerning the three topics reported below:

1. Physician-patient interaction for MS progression communication
2. Biomarkers for early MS progression identification
3. Diagnostic symptoms and tools for early MS progression identification

**Fig. 1:**
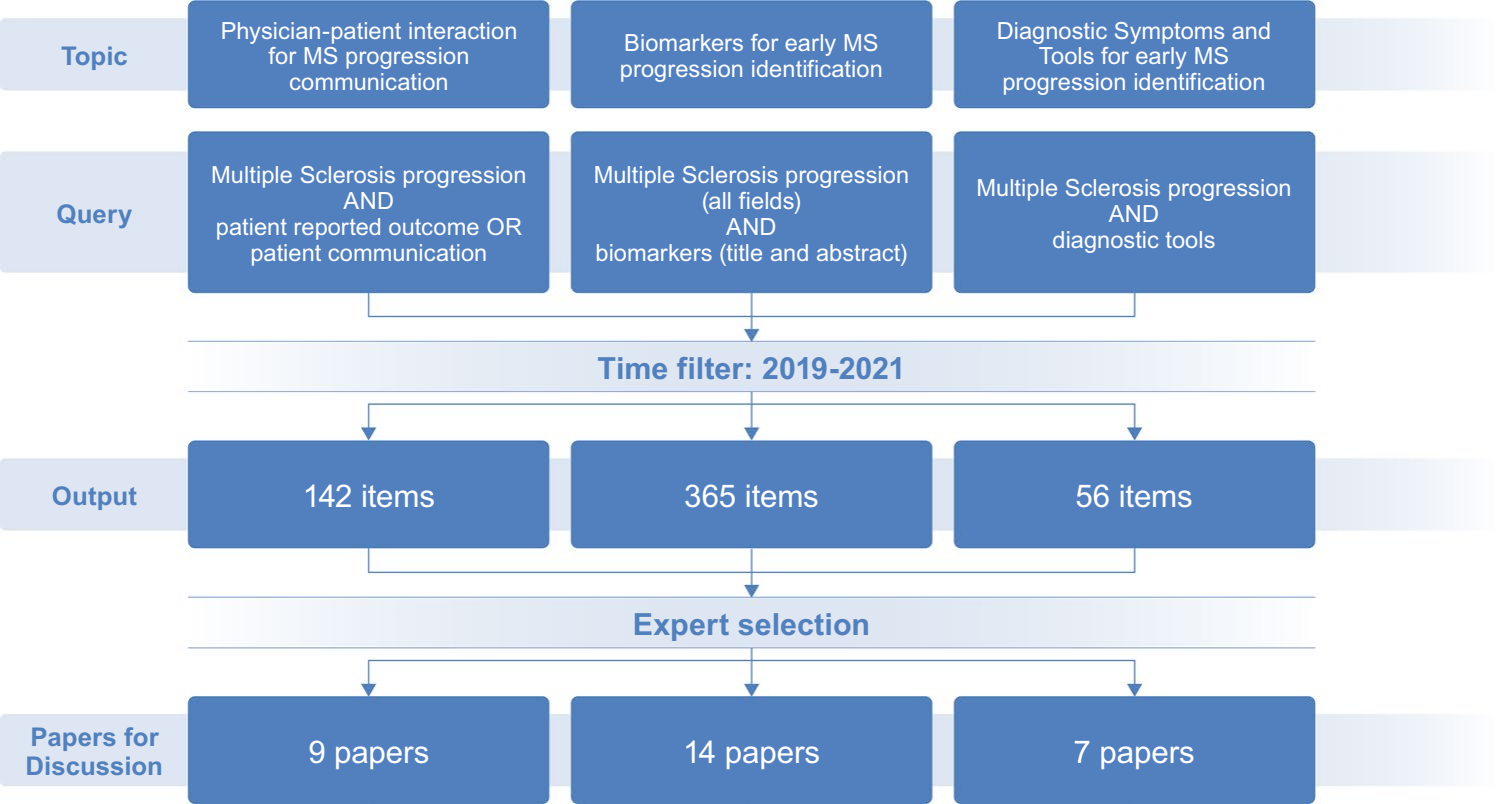
Flow chart followed for the identification of the pertinent literature to be discussed by the panel of experts

Literature search identified 142 items for the first query, 365 and 56 for the second and third, respectively. Participants were assigned to one of the three groups, and each one selected around 10 articles to be used as a starting point for their discussion within the group. The flow chart followed for paper selection is reported in Fig. 1.

During the first meeting, the current state of the art was presented. The experts discussed the three topics, highlighting possible solutions to the gaps identified. The result of the discussion was shared in plenary session at the end of the first meeting. On the basis of the output of the first meeting, one of the organizers devised three statements for each topic, on a 4-level Likert scale, to be administered to all the participants.

In the second meeting, the panel reviewed the proposed solutions and undertook further discussion on the statements in plenary session, to find agreement on actions to be applied in clinical practice. This methodology allowed an extensive and productive discussion among the expert panel.

The outcome of the expert panel is summarized as follows, with pinpointed sentences, percentage of agreement on each sentence, and main discussion points.

## Physician–patient interaction for progression communication

**Fig. 2:**
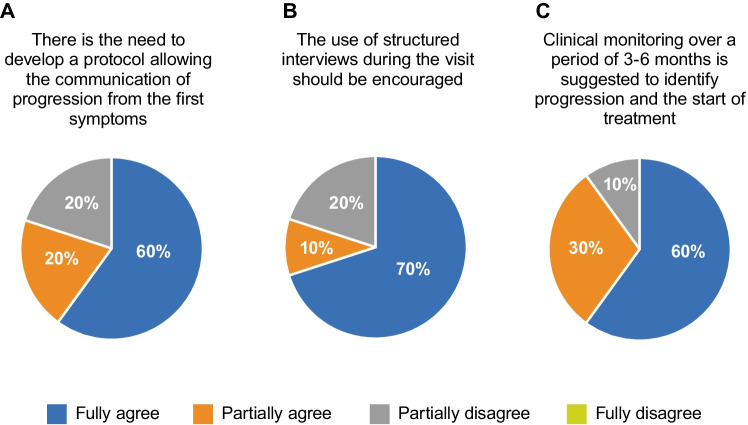
Experts’ agreement on physician–patient interaction for progression communication issue. The panel of experts discussed three sentences with the respective level of agreement

1.1 Panelists underlined the need to develop a standardized shared protocol allowing the timely communication of conversion from RRMS to SPMS/progressive MS as it begins (Fig. 2A).

(A) The main points to be considered when drafting the protocol were as follows:

- The need to increase patient awareness, both of the possibility of disease conversion to progression and on the existence of an appropriate treatment. Patients should be aware of the importance of regular disease monitoring and reassessment, to allow the timely adjustment of the therapy, which will result in delaying disease progression and tackling the accumulation of disability. Postponing facing the topic of progression reduces patient engagement. The HCP has to be clear in explaining the therapy options for the progressive phase, as the patient may see the possibility of progression as a limit to reaching and maintaining a good quality of life (QoL).
- The patient should be informed about progression only by the MS specialist to avoid independent confounding sources of information. Follow-up visits are the right time to introduce the topic.
- Earlier awareness may create anxiety, discouragement, and lack of adherence towards therapy. Thus, it is mandatory that HCPs pay great attention to each patient’s personal feelings, attitudes and psychological status, and their need for information. Sensitive communication, the use of a reassuring tone, and the right timing of information delivery should be carefully considered to allow patient awareness and engagement.
- The patients appreciate telemedicine visits and the use of digital devices as a communication tool.

1.2 The use of periodic structured interviews should be encouraged to get insights into the patients’ perspective and functional areas mainly associated with SPMS, with the aim of identifying patients at risk of progression (Fig. 2B). Interviews are helpful since the following:

- The first signs of progression are often detected by the patient, even before clinical and paraclinical evidence. Structured interviews help to investigate and understand the patient’s overall QoL. Patient-reported outcomes, digital tools, and digital visits could help identify the progression early, providing insights on aspects that usually do not come to the attention of the HCP (such as a change in everyday activities).
- The interview is a rapid tool, allowing the acquisition of information without the need for an extended visit. All the information should be integrated with the expertise of the HCP.

1.3 Clinical monitoring over a period of 3–6 months is suggested to identify and confirm progression and to discuss treatment options (Fig. 2C):

- Transition to disease progression is very variable among patients. Nevertheless, slight progression of signs and symptoms evaluated and confirmed after 3–6 months may identify the patient at risk. Progression should be clearly discriminated from the worsening associated with relapses that can still characterize SPMS. The impact of confounding factors like seasonality should also be taken into account.
- Symptoms that slowly get worse over 3–6 months can be considered red flags; if they persist up to 6–12 months, progression can be confirmed and appropriate therapy initiated. Signs of recent (clinical and/or radiological) disease activity are currently mandatory for prescription of new drugs.

Observation of slow worsening of symptoms can be facilitated by telemedicine, which allows more frequent visits and is appreciated, especially by patients with reduced mobility. The use of digital tools for monitoring progression (e.g., eSDMT) should be encouraged. A wrist-held dynamometer may help to get an objective insight into motor capabilities, although an improvement in the detecting ability of the device is wanted.

## Biomarkers for progression identification (clinical and paraclinical)

**Fig. 3:**
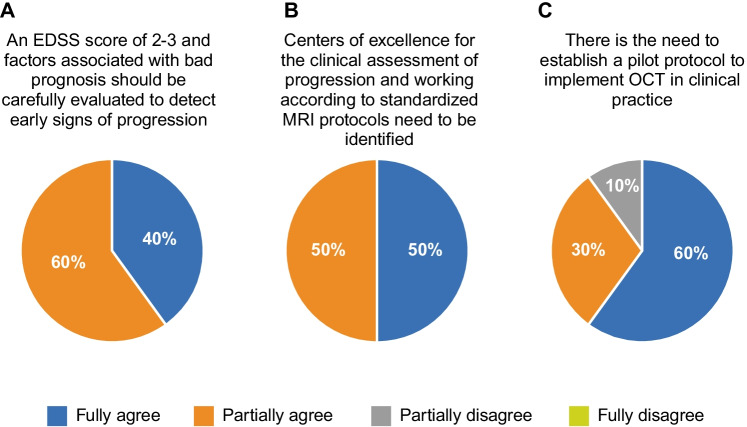
Experts’ agreement on the need for biomarkers for progression identification (clinical and instrumental). The panel of experts discussed three sentences with the respective level of agreement. EDSS, Expanded Disability Status Scale; MRI, magnetic resonance imaging; OCT, optical coherence tomography

2.1 Patients with EDSS of 2.0/3.0 and factors associated with bad prognosis should be carefully evaluated to detect early signs of progression (Fig. 3A). Experts agree on the following points:

- EDSS cutoff at values of 2.0/3.0 could be too low to correctly identify patients at risk of progression. The EDSS score cutoff should be higher (3.0/4.0).
- There is the need to validate and implement clinical indicators that may predict progression (disability, cognition, fatigue, sexual and sphincteric disturbances). Guidelines on the assessment of cognitive functions in MS are urgently needed.
- There is a need to focus on prognostic markers identifying the transition.
- A “model of progression” for the disease could be elaborated based on data coming from the first 2–5 years of the disease.
- The use of neurofilaments as biomarkers needs to be validated as the methods of analysis are not standardized for clinical practice.

2.2 Centers of excellence for the clinical assessment of progression and working according to standardized MRI protocols need to be identified (Fig. 3B):

- MRI is an easily accessible tool, and its use should be implemented and standardized among specialized MRI centers equipped to evaluate brain atrophy. The increase in brain atrophy without inflammatory activity [71] and progression independent of relapse activity (PIRA) could herald progression [72]. Difficulties in standardization of MRI acquisition and post-processing make identifying signs of progression unreliable. The current conventional MRI protocols do not always allow early and reliable identification of markers associated with transition to SPMS (e.g., increasing number/volume of brain white matter lesions, high/increasing number of cortical lesions, accelerated rate of brain global/regional atrophy, high/increasing number of slowly expanding lesions, and accelerated rate of spinal cord damage/atrophy).
- MRI acquisition and processing procedures need to be standardized, and shared high-qualified MRI centers would reduce the possibility of biases. It is necessary to identify at regional or inter-regional level one or more referral centers for the standardized evaluation of MRI biomarkers suggestive of progression. The center should be equipped with a 3 T MRI scanner.
- Neurologists and neuroradiologists should define standard criteria and procedures, and a committee/consortium should create a list of certified radiological centers, to reach standardization of protocols and equipment at a regional level. The patient should be provided with such a list. Patient MRI evaluation by a referral center would be required every 1–2 years.
- All the MS centers should be provided with a univocal protocol to be followed for MRI analysis; the results could then be analyzed by a unique MS referral center. Economic barriers in some areas and an overall shortage of personnel also need to be considered.

2.3 The panel of experts suggests establishing a pilot protocol to implement OCT in clinical practice (Fig. 3C):

- Biomarkers such as RNFL and GCIP can be measured by OCT. They seem to be promising and sensitive markers of progression, even if they are characterized by a certain degree of interindividual variability that needs to be taken into account. It is mandatory to follow the univocal standardized protocol that identifies parameters to be shared among MS centers to implement this measure in routine clinical practice along with MRI [73].
- OCT could be a good screening test for all patients and not only those at risk of progression.
- Other promising biomarkers useful to evaluate progression are Nfl, CHI3L1, PET radiotracers, and MRI biomarkers. Univocal protocols for their quantification are needed, because they show high variability, and their reliability can be affected by potential bias.

## Diagnostic symptoms and tools

**Fig. 4:**
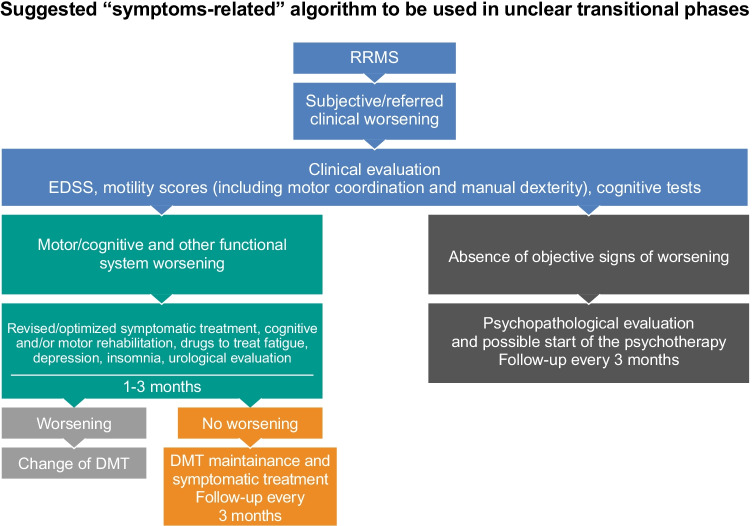
Suggested “symptom-related” algorithm to be used in clinical practice to evaluate unclear diagnostic transitional phases. DMT, disease-modifying treatment; EDSS, Expanded Disability Status Scale; RRMS, relapsing–remitting multiple sclerosis

3.1 The panel suggests using a symptom-related algorithm to identify patients at risk of progression (Fig. 4).

**Fig. 5:**
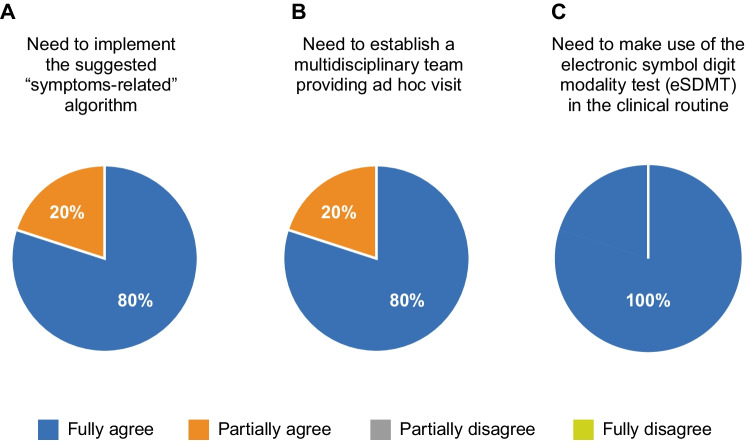
Experts’ agreement on the strategies for diagnostic symptoms and tools. The panel of experts discussed three sentences with the respective level of agreement

- This algorithm could be of help in clinical practice, and there is a good agreement among the experts about its use (Fig. 5A). The algorithm summarizes a possibly useful strategy to identify patients facing the transition period: an early and close evaluation of RRMS patients who refer with a worsening condition, through cognitive tests, EDSS, and other disease scores. In this scenario, anamnesis needs to be improved. A follow-up every 3–6 months is scheduled if no worsening is detected. This increased frequency, as opposed to the standard 6–12 month follow-up visits, aims to avoid delay in identifying progression. According to the algorithm, a comprehensive neuropsychiatric assessment is suggested for each patient.
  When signs of worsening are detected, HCPs should verify and modulate the symptomatic treatment. If no improvements are registered after 1–3 months, a DMT switch is suggested with subsequent follow-up visits every 3–4 months.
  The proposed algorithm may represent an objective tool to identify patients at risk of progression by closely monitoring the first signs of worsening and verifying whether modulation of symptomatic therapy results in a short period of time. It is mandatory to have objective criteria for the identification of SPMS, because a discrepancy between physicians could result in an inappropriate and inconsistent classification of patients’ phenotype with detrimental consequences on treatment choices and enrollment in clinical studies.

3.2 To improve disease management and treatment, there is a strong need for ad hoc visits with the multidisciplinary team and the routine use of a cognitive screening test (Fig. 5B and C):

- The professionals included in a representative multidisciplinary team are neurologists, neuroradiologists, ophthalmologists, neuropsychologists, psychiatrists, and MS-specialized nurses. They collaborate by collecting data about different functional areas, allowing a complete anamnesis and the evaluation of the overall neurologic status of the patient. Other figures that should be involved are caregivers and rehabilitation specialists. Multidisciplinary teams across Europe are differently composed: in Germany and the UK, besides specialized neurologists, physiotherapists and rehabilitation specialists are involved; social workers are present in France and the UK [74].
- Patients at risk of progression would benefit from an extended visit time, where different needs would be better evaluated.
- A medical office with an MS specialist dedicated only to transitioning and SPMS patients is suggested. Here, the multidisciplinary team could periodically reassess the patient’s symptoms dedicating more time to the SPMS patient to overcome their reluctance in communicating signs of worsening. High attention has to be paid to fatigue, for which an objective scale is still lacking, and to upper limb dexterity. Tools such as eSDMT, MS Pro Discuss, and CogEval could help. In particular, CogEval should be evaluated in light of the sociocultural context of the patient. Should the dedicated office be absent, the MS specialists must request a specific evaluation by other specialists belonging to the multidisciplinary team, according to the specific patient’s condition.
- The nurses may have a prominent role in identifying the first signs of transition, as they closely monitor the patients and meet them more often [75].
- The caregiver should be strongly engaged in the disease management path. The role of the caregiver is central in understanding the signs of progression. The caregiver is aware of the patient’s everyday life and has a whole vision of the patient situation, which is extremely useful to clarify the scenario of symptoms and their evolution.
- Telemedicine is another central aspect that needs to be implemented. Telemedicine is usually appreciated by patients with reduced mobility and should integrate traditional visits of follow-up, ensuring closer monitoring of patients.
- The SDMT through a smartphone app is a useful and fast tool, showing comparable reliability to the SDMT administered by the HCPs, and would be of great help to detect signs of progression and evaluate the success of the therapy. An increase of 4 points in the SDMT is considered clinically relevant. The MS professionals pointed out that the app needs to be translated into the local language, and that it is important to get a graphical output of the analysis provided by the app so that the physician can quickly read the results.

## Summary

To summarize the joint work of the expert panel, these are the core points that were identified as central for the management of patients with MS experiencing the first suspected symptoms of progression:

- MS specialists, general practitioners, caregivers, and nurses should cooperate to increase the patient’s awareness on his/her condition and on the DMT for RRMS in order to prevent or postpone the development of SPMS.
- During follow-up visits, the possibility of progression and DMT switch should be discussed. The patient should pay attention to the evolution of the disease and comply with increased frequency in monitoring and with RRMS therapy. Patients that are suspected to be moving into the transition phase should be closely monitored for 3–6 months to avoid delay in SPMS identification and treatment.
- On identifying SPMS, the MS specialists have to clearly explain the scenario to their patients. The biomarkers of progression should be evaluated to identify the degree of evolution of the disease, and nurses and caregivers should cooperate in highlighting symptoms and clarifying disease definition. The identification of new biomarkers would help to define the evolution of the disease better, and MS specialized centers have to be identified to conduct the appropriate examination of the biological and radiological markers associated with progression.
- DMT decision requires an experienced team which would assess the patient’s condition and start the appropriate treatment; adherence with the new treatment is expected to be high, given the type of administration (oral route) and the available reimbursement from the National Health Service.
- Follow-up visits should be frequent and based on examinations by the multidisciplinary team, so that different aspects of the disease are carefully evaluated. Digital apps and telemedicine would support patient monitoring. Patients and their caregivers should be given support to improve daily QoL.
- A reassessment of the patient’s condition should be provided every 6 months to evaluate the efficacy of the therapy. As the treatment for SPMS has been recently approved, updating guidelines for reassessment and possible adjustments must be ongoing.

## Conclusion

SPMS is poorly identified, and its incidence and prevalence are underestimated in Italy, owing to the lack of reliable diagnostic tools. Thus far, the lack of a specific, effective, and safe treatment discouraged MS professionals from a timely and clear communication of the transition phase to the patient, even after the progression to SPMS was confirmed. Now that a therapeutic option is available, there is the need for adequate education both for patients, regarding the evolution of the disease and the possibility of a specific treatment, and for MS professionals, to train them to interact with the patients effectively and sensitively. The use of digital tools to detect signs of progression and support the patients during the disease course must be increased. Earlier communication means earlier identification of progression, which accounts for early and more effective treatment.

Early active SPMS may represent an important therapeutic window where early treatment has been shown to increase the time taken to reach higher disability milestones, finally preserving the patient’s QoL.
